# IUC24025-78 Early results of moderate hypofractionated radiotherapy implementation for localized prostate cancer patients in Vietnam

**DOI:** 10.1093/oncolo/oyaf248.033

**Published:** 2025-08-29

**Authors:** C Nguyen Dinh, K Nguyen Xuan, T Pham Quang, D Le Manh, B Bui Quang

**Affiliations:** 108 Military Central Hospital, Vietnam; 108 Military Central Hospital, Vietnam; 108 Military Central Hospital, Vietnam; 108 Military Central Hospital, Vietnam; 108 Military Central Hospital, Vietnam

## Abstract

**Background:**

To evaluate the early outcomes of moderately hypofractionated radiotherapy in patients with localized prostate cancer.

**Methods:**

A prospective, single-institution study was conducted on 41 prostate cancer patients with stage T1-3bN0M0 who underwent radical radiotherapy with a total dose of 60 Gy delivered in 20 fractions combined with androgen deprivation therapy. The Kaplan–Meier curve was used to estimate biochemical failure and survival rates. Side effect was evaluated according to the CTCAE 5.0 criteria.

**Results:**

The median follow-up time was 38 months. The 4-year biochemical relapse-free survival, disease-free survival, and overall survival were 93.5%, 85,7%, and 93.8%, respectively. Early gastrointestinal (GI) and genitourinary (GU) system side effects of grade 1-2 were 34.0% and 29.8%, respectively. No early side effects were grade 3 or higher. The late complication was observed in one patient who developed grade 3 rectal inflammation.

**Conclusions:**

Moderate hypofractionated radiotherapy in localized prostate cancer showed excellent survival and a low rate of serious side effects.

